# Perioperative transcutaneous auricular vagus nerve stimulation and EEG biomarkers of sleep improvement after gynecologic laparoscopy: protocol for a randomized controlled trial

**DOI:** 10.3389/fmed.2026.1880911

**Published:** 2026-08-07

**Authors:** Xun-qi Huang, Long-xin Zhang, Li-hua Zheng, Zhenna Wang, Wen-shui Yao, Qiu-ying Wang, Jing Wang

**Affiliations:** 1 Department of Anesthesiology, Fujian Maternity and Child Health Hospital, College of Clinical Medicine for Obstetrics & Gynecology and Pediatrics, Fujian Medical University, Fuzhou, Fujian, China; 2 Department of Anesthesiology, The Quzhou Affiliated Hospital of Wenzhou Medical University, Quzhou, Zhejiang, China; 3 Department of Obstetrics and Gynecology, Fujian Maternity and Child Health Hospital, College of Clinical Medicine for Obstetrics & Gynecology and Pediatrics, Fujian Medical University, Fuzhou, China

**Keywords:** electroencephalography, gynecologic surgical procedures, postoperative sleep disturbance, randomized controlled trial, transcutaneous auricular vagus nerve stimulation

## Abstract

**Background:**

Postoperative sleep disturbance (POSD) affects up to 62% of patients undergoing gynecologic laparoscopy and is associated with prolonged recovery, chronic pain, and cardiovascular complications. Transcutaneous auricular vagus nerve stimulation (taVNS) modulates thalamocortical oscillations through afferent vagal pathways, offering a potential non-pharmacological mechanism to prevent acute sleep disruption during critical perioperative neuroplastic windows. This study aims to evaluate whether perioperative dual-session taVNS reduces the incidence of clinically significant POSD compared with sham stimulation.

**Methods:**

This single-center, randomized, double-blind, sham-controlled trial is planned to enroll 116 patients aged 18–65 years scheduled for elective gynecologic laparoscopy. Participants will be allocated 1:1 to active taVNS or sham stimulation using permuted block randomization with concealed allocation. Blinding is maintained for participants, anesthesia providers, surgeons, postoperative ward staff, outcome assessors, and data analysts. Active taVNS will be applied to the left cymba conchae (20 Hz, 250 μs) in two sessions: 30 min before anesthesia induction and 5 min after extubation. The sham stimulation current amplitude will be incrementally increased until the participant perceives a tingling sensation; the current will then be immediately discontinued. Each session will last 30 min with continuous frontal electroencephalography (EEG) monitoring (0.5–45 Hz) at six timepoints. The primary endpoint is the incidence of POSD on postoperative day 1 (AIS score ≥ 6). Secondary endpoints include sleep quality trajectory (AIS, actigraphy), anxiety and depression (HADS), recovery quality (QoR-15), and exploratory EEG spectral analyses.

**Discussion:**

This trial is the first to introduce a perioperative dual-session taVNS protocol integrated with continuous EEG monitoring, investigating its preventive effects on acute POSD in gynecologic laparoscopic patients through a comprehensive set of mechanistic and clinical endpoints. Comprehensive multidimensional assessment combines subjective, objective, and mechanistic (EEG spectral analysis) measures. If proven effective, taVNS could offer a safe and cost-effective non-pharmacological adjunctive therapy for managing POSD in the perioperative setting. Furthermore, the findings have the potential to elucidate how perioperative cortical oscillatory dynamics regulate postoperative sleep quality, offering mechanistic insights for future neuromodulation interventions.

**Clinical trial registration:**

http://www.chictr.org.cn, identifier ChiCTR2600120222.

## 1 Introduction

Postoperative sleep disturbance (POSD) is observed in up to 62% of patients undergoing gynecologic laparoscopy, representing a substantial clinical burden (1, 2). This condition typically manifests on the first postoperative night and may persist for several days, significantly contributing to serious complications including chronic pain (3), delirium (4), cardiovascular events (5), and delayed recovery in Enhanced Recovery After Surgery (ERAS) pathways (6). The pathophysiology of POSD may be attributed to a multifactorial etiology, encompassing the disruption of the sleep-wake cycle due to the administration of general anesthetics, in conjunction with inflammation induced by surgical trauma and heightened sympathetic activity (7). These factors collectively contribute to postoperative reductions in rapid eye movement (REM) sleep, slow-wave sleep, and result in sleep fragmentation (5).

Current mainstay treatments rely primarily on pharmacological interventions such as dexmedetomidine (8), melatonin, and zolpidem (9); however, their routine use is limited by adverse effects including respiratory depression, hypotension, bradycardia, and delirium (10). Non-pharmacological interventions such as earplugs, eye masks, relaxation training, music therapy, and aromatherapy have been attempted to improve POSD (11). However, the efficacy of these methods varies considerably, and the quality of available evidence is generally low. In recent years, brain function modulation techniques, as emerging non-invasive neuromodulation approaches, have shown potential in ameliorating POSD. Du et al. found in a randomized, double-blind, sham-controlled trial that a single session of prefrontal transcranial alternating current stimulation significantly reduced the incidence of POSD on the first day after gynecological laparoscopic surgery (12). Nevertheless, this method still has certain limitations, including high equipment cost, complex operation procedures, and possible adverse reactions, which limit its widespread clinical adoption. Consequently, there is a critical demand for therapies that are safe, effective, well-tolerated, and easily implemented to enhance postoperative sleep quality.

Transcutaneous auricular vagus nerve stimulation (taVNS) is a non-invasive neuromodulation approach that delivers electrical pulses to auricular branches of the vagus nerve. By stimulating vagal afferent fibers, taVNS engages brainstem–autonomic pathways (including projections to the locus coeruleus) and modulates thalamo–cortical activity, which may shift cortical arousal dynamics and thereby improve sleep–wake regulation, particularly during perioperative neuroplastic windows. Consistent with this mechanism, prior studies suggest taVNS may reduce POSD (13, 14). Randomized sham-controlled trials demonstrate that taVNS significantly improves sleep quality scores, enhances slow-wave sleep continuity, and boosts daytime functioning in patients with chronic insomnia (15, 16). Prior research indicates that taVNS modulates EEG power spectral density by mitigating reductions in delta power and increases in alpha/beta power associated with negative emotional states (17), with significant effects observed in the prefrontal region (18). Molefi et al. demonstrated that taVNS modulates EEG theta spectral power during visually induced motion sickness, a negative physiological state (19). These findings suggest that taVNS may restore cortical electrophysiological homeostasis, providing a potential neurophysiological basis for sleep improvement. Despite evidence that taVNS modulates EEG patterns and that perioperative EEG characteristics are predictive of POSD, it is unclear whether taVNS delivered can effectively modulate EEG changes to prevent acute sleep disruption.

This protocol describes a single-center randomized, double-blind, sham-controlled trial evaluating whether a dual-session taVNS regimen administered at two perioperative timepoints–30 min before anesthesia induction and 5 min after extubation–reduces the incidence of clinically significant POSD on postoperative day 1 (AIS ≥ 6). Active taVNS will be applied to the left cymba conchae using a predefined stimulation parameter set, while sham stimulation uses an identical device and blinded sensory masking procedures. Continuous frontal EEG will be acquired during the stimulation periods to enable predefined analyses of taVNS-related spectral modulation and to explore EEG biomarkers associated with subsequent sleep outcomes.

## 2 Methods

### 2.1 Study design

**FIGURE 1:**
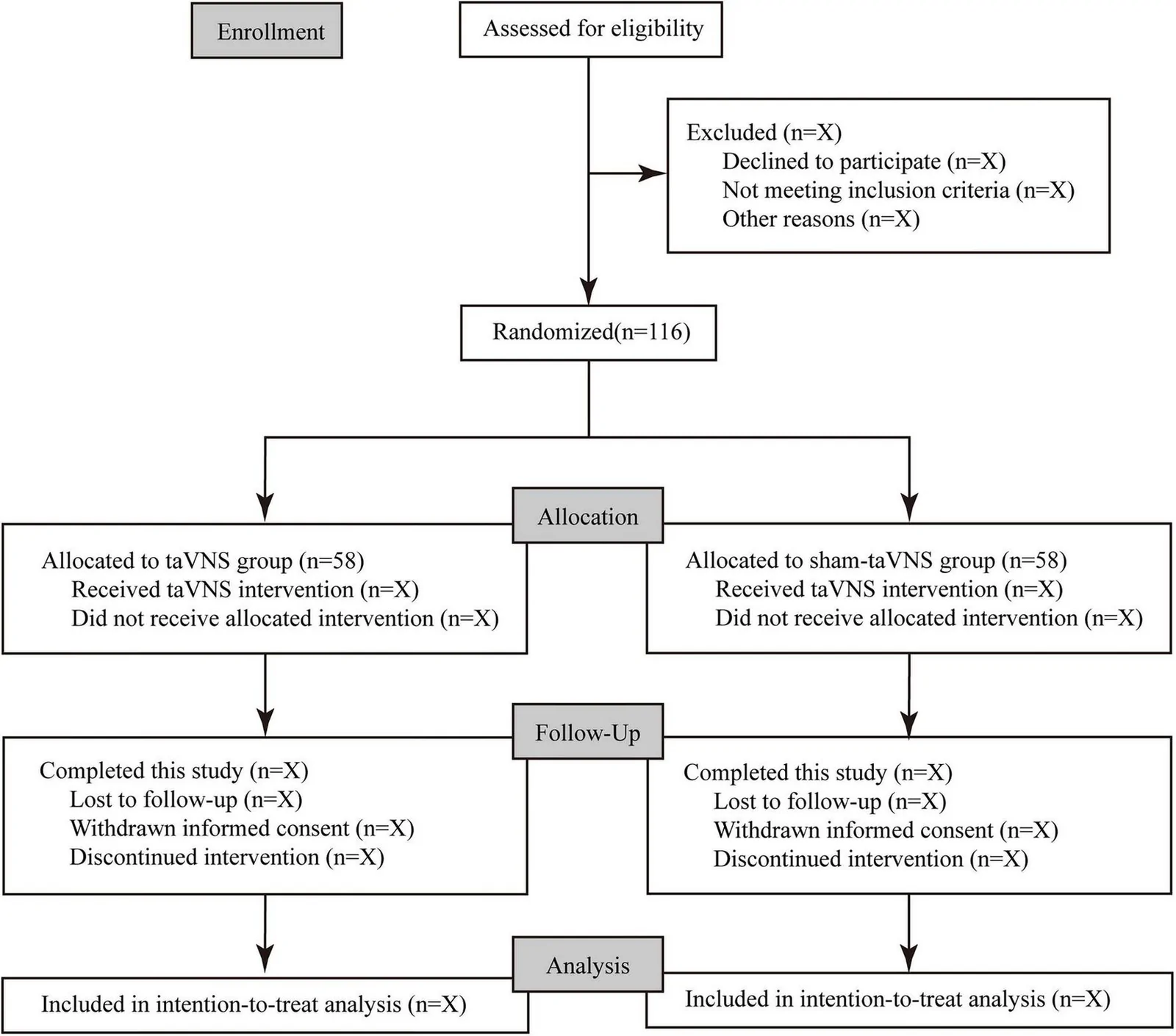
Flowchart of the study design.

This single-center, prospective, randomized, double-blind, sham-controlled trial will be conducted at Fujian Maternity and Child Health Hospital. A total of 116 participants will be enrolled for elective gynecologic laparoscopic surgery under general anesthesia. The Ethics Committee of Fujian Maternity and Child Health Hospital approved this protocol (approval number 2026KY074), and it is registered with the Chinese Clinical Trial Registry (ChiCTR2600120222). Participants will be thoroughly informed about the study procedures and will provide written consent before enrollment. The protocol is reported in accordance with the SPIRIT 2013 statement. A flowchart illustrating participant flow through recruitment, interventions, and assessments is presented in Figure 1. The schedule of events is presented in Table 1.

**TABLE 1:** Study schedule for data measurements.

Study period											
	Enrollment	Allocation	Post allocation								
Timepoint	Pre1	0 d	T0	T1	T2	T3	T4	T5	POD 1	POD 2	POD 3
Enrollment											
Eligibility screen	X										
Informed consent	X										
Random allocation		X									
Interventions											
Active taVNS				X				X			
Sham taVNS				X				X			
Assessments											
Baseline data	X	X									
EEG			X	X	X	X	X	X			
AIS	X								X	X	X
HUAWEI WATCH GT2	X								X	X	X
QoR-15	X								X		
NRS	X								X		
PSQI	X										
HADS-A	X								X	X	X
HADS-D	X								X	X	X
Adverse events	Record at any time at any time at any time										

Pre1 for preoperative day 1, T0, preoperative baseline (5 min before taVNS); T1, preoperative sustained stimulation period (20–30 min taVNS); T2, post-induction (5 min after intubation); T3, intraoperative maintenance (20 min after incision); T4, peri-extubation (end of surgery); T5, postoperative sustained stimulation (20–30 min post-taVNS). POD 1–POD 3 for postoperative days 1–3; taVNS, transcutaneous auricular vagus nerve stimulation; EEG, electroencephalography; AIS, Athens Insomnia Scale; QoR-15, 15-item Quality of Recovery questionnaire; NRS, numeric rating scale; PSQI, Pittsburgh Sleep Quality Index; HADS-A/HADS-D, Hospital Anxiety and Depression Scale-Anxiety/Depression subscale.

### 2.2 Participants

Potential participants will be screened for eligibility during the preoperative assessment, typically scheduled 1 day prior to surgery. Those meeting eligibility criteria will receive detailed written and verbal study information, have the opportunity to ask questions, and provide written informed consent. Participation is voluntary, and individuals may withdraw at any time without impacting their standard clinical care.

#### 2.2.1 Inclusion criteria

Participants will be eligible if they are aged 18–65 years, classified as ASA physical status I–II, have a BMI between 18 and 30 kg/m^2^, and be scheduled for elective gynecologic laparoscopic surgery expected to last at least 60 min. Additionally, they must be proficient in Mandarin Chinese with the ability to provide written informed consent.

#### 2.2.2 Criteria for exclusion

(1) Broken skin, infection, or inflammation of the external ear or auditory canal; history of tinnitus, Meniere’s disease, or cardiac arrhythmia with symptoms; (2) Presence of an implanted cardiac pacemaker, defibrillator, or other active implantable device; (3) History of neuropsychiatric disorders, such as depression, anxiety, epilepsy, or schizophrenia; (4) Severe hepatic impairment (Child-Pugh class B or C) or renal impairment (eGFR < 60 mL/min/1.73 m^2^ as per the CKD-EPI equation); (5) Planned postoperative ICU admission; (6) Pregnancy or breastfeeding.

#### 2.2.3 Dropout criteria

(1) Occurrence of serious adverse events attributed to the intervention or surgical procedure; (2) voluntary withdrawal by the participant; (3) intraoperative conversion to open surgery or unplanned admission to the ICU; and significant deviations from the study protocol affecting primary outcome assessment.

### 2.3 Randomization and blinding

An independent statistician will use SPSS (version 26.0, IBM Corp.) to create the allocation sequence through permuted block randomization with randomly varying block sizes of 4 and 6, ensuring balanced allocation throughout the enrollment periods. Participants will be randomly allocated to either active taVNS or sham stimulation in a 1:1 ratio. The statistician will prepare sequentially numbered, sealed, opaque envelopes with group assignments, which will be stored in a locked cabinet accessible solely to a designated unblinded device coordinator. This coordinator responsible exclusively for device preparation will open the envelopes and configure the devices in the pre-anesthesia holding area. Following device connection, the coordinator will exit and have no further contact with participants, clinical care, or outcome assessments.

Throughout the trial, group allocation will remain concealed from anesthesia providers, surgeons, outcome assessors, postoperative ward staff, participants, and data analysts. To ensure participant blinding, identical electrode placement and device appearance will be used for both groups. All devices will display identical interface indicators regardless of stimulation status. Participants will be informed that “both interventions involve ear stimulation with variable mild sensations; individual perception does not indicate assignment.” To maintain assessor blinding, the unblinded device coordinator will ensure that the device interface remains concealed from clinical staff and will not participate in any outcome assessments. Unblinding will be permitted only for serious adverse events that require knowledge of allocation for clinical management. The principal investigator will hold the master randomization key in a sealed, signed envelope. All unblinding events will be recorded along with their reasons and timestamps.

Blinding integrity will be assessed using Bang’s Blinding Index (BI) (20) at two time points: immediately following the second stimulation session and on POD 3. Bang’s Blinding Index for each arm = (correct guesses − incorrect guesses)/(total participants in that arm), ranging from −1 to 1, with 0 indicating perfect blinding. A two-sided 95% confidence interval for BI will be constructed for each arm. If the interval excludes 0 for either arm, indicating statistically significant deviation from random guessing, blinding will be deemed inadequate, and sensitivity analyses will compare primary outcomes with and without potentially unblinded participants.

### 2.4 Anesthesia protocol

Standard monitoring will include electrocardiography, pulse oximetry, and noninvasive blood pressure. General anesthesia will be initiated using intravenous administration of propofol (2 mg/kg), sufentanil (0.5 μg/kg), and rocuronium (0.6 mg/kg). Mechanical ventilation will be administered in volume-controlled mode with an inspiratory to expiratory ratio of 1:2, a respiratory rate of 10–12 breaths per minute, and a tidal volume of 6–8 mL per kg of predicted body weight. End-tidal CO_2_ levels will be kept between 35 and 45 mmHg. Anesthesia will be sustained using continuous infusions of remifentanil (0.05–0.15 μg⋅kg^–1^⋅min^–1^) and propofol (50–100 μg⋅kg^–1^⋅min^–1^), supplemented with intermittent rocuronium boluses (0.2 mg⋅kg^–1^) as needed. The ConView anesthesia consciousness monitor (Model YY-105, Zhejiang Puke Medical Technology Co., Ltd., China) will be utilized to evaluate anesthetic depth. This apparatus produces the anesthesia consciousness index (Ai), a quantitative metric derived from the analysis of frontal EEG signals. The Ai scale spans from 0, representing the deepest level of anesthesia, to 100, indicating full wakefulness. This scale exhibits high reliability and a significant correlation with the bispectral index (BIS) (21). Throughout the surgical procedure, the anesthesia consciousness index (Ai) will be maintained within the range of 40–60. Extubation will be performed when patients demonstrate regular spontaneous respiration, adequate minute ventilation, and the ability to follow commands. Patients are transferred to the post-anesthesia care unit (PACU) when their Steward Recovery Score is 5 or above. Upon arrival in the PACU, patient-controlled analgesia (PCA) will be initiated using a consistent regimen: sufentanil at a dosage of 1.5 μg/kg combined with tropisetron 6 mg in 100 mL of normal saline, administered via an electronic PCA pump. The pump settings will be programmed as follows: a continuous background infusion rate of 2 mL/h, a PCA bolus dose of 1 mL, and a lockout interval of 15 min. Rescue analgesia will be standardized such that patients with a Numeric Rating Scale (NRS) pain score greater than 5 at rest will receive intravenous flurbiprofen 50 mg as an additional analgesic. To minimize environmental confounding, all participants will be admitted to a single, dedicated gynecology ward unit following surgery. This unit features uniform room configurations (two beds per room, standardized layout), consistent lighting protocols (lights out at 22:00, dimmed night lighting), and noise control policies (designated quiet hours 22:00–06:00). The same nursing team, trained in ERAS protocols, will provide postoperative care to all participants, ensuring consistency in care delivery, visitor management, and nighttime disturbance patterns.

### 2.5 EEG power spectrum processing

Frontal quantitative EEG parameters will be obtained using the ConView YY-105 anesthesia depth monitoring system, employing bilateral frontal electrodes at a sampling rate of 500 Hz. EEG power spectral density will be estimated using the multitaper method (15 tapers, time-half bandwidth product = 8) with a 4-s window length and 50% overlap (2-s step), implemented in MATLAB’s pmtm function. The bandpass filter (0.5–45 Hz) will be applied as a zero-phase filter using forward-reverse filtering to avoid phase distortion. Artifact-free epochs will be extracted at six predetermined time points: T0 (pre-stimulation baseline): 5 min prior to the first taVNS session; T1 (preoperative sustained stimulation), 20–30 min following the initiation of taVNS; T2 (post-induction), 5 min after tracheal intubation; T3 (intraoperative maintenance), 20 min subsequent to the skin incision; T4 (peri-extubation), at the conclusion of surgery, immediately preceding emergence from anesthesia; and T5 (postoperative sustained stimulation), 20–30 min after the initiation of postoperative taVNS.

### 2.6 Study interventions

The interventions will be administered at two specific time points: 30 min before the induction of anesthesia and 5 min after tracheal extubation in the PACU, with each session lasting 30 min. Prior to the initiation of stimulation, the left auricle will be cleansed using gauze moistened with a saline solution. The unblinded coordinator will position the taVNS device (model taVNS501, Reshen Medical Technology Co., Ltd., Changzhou, China) according to the randomization assignment.

Participants assigned to the taVNS group will undergo stimulation at the left cymba conchae, an area innervated by the auricular branch of the vagus nerve. This stimulation will be administered using a pulse frequency of 20 Hz and a pulse width of 250 μs. The stimulus amplitude will initially be set to zero and will be gradually increased until the participant experiences a tolerable tingling sensation, typically within 30 s, which will be maintained throughout the 30-min session. In the sham group, the electrode placement will be identical to those of the active taVNS group using the same stimulation parameters. The stimulus amplitude will be incrementally increased until the participant perceives a tingling sensation after which the current will be immediately discontinued. The device will remain powered on, with identical auditory and visual cues, for the entire 30-min session to ensure the absence of sustained neuromodulatory effects while preserving participant blinding.

During each stimulation, participants will remain in a supine position while undergoing continuous monitoring through electrocardiography, pulse oximetry, and noninvasive blood pressure assessment. Trained personnel will provide constant observation of the participants. Stimulation will be immediately stopped if any of the following occur: (1) unbearable discomfort; (2) oxygen saturation (SpO_2_) drops below 90%; (3) heart rate falls below 50 beats per minute or decreases by more than 20% from the initial rate; (4) a new arrhythmia begins; (5) hemodynamic instability, defined as systolic blood pressure below 90 mmHg or above 180 mmHg; or (6) any significant adverse event. The attending physician will decide on the suitable clinical management.

### 2.7 Outcomes

#### 2.7.1 Main outcome

The primary endpoint is the incidence of POSD on POD 1, defined as an Athens Insomnia Scale (AIS) score ≥ 6. The AIS is a validated eight-item instrument measuring nocturnal sleep parameters and daytime functioning, with total scores ranging from 0 to 24 (22).

#### 2.7.2 Secondary outcomes

1. Trajectory of Postoperative Sleep Quality (1) Subjective sleep quality: The trajectory of AIS total scores from Preoperative Day 1 (Pre-1) through POD 3. (2) Objective sleep parameters: Wrist actigraphy (HUAWEI WATCH GT2) measured continuously from Pre-1 through POD 3. Metrics include sleep continuity (latency, awakenings, wake after sleep onset, efficiency, and total sleep time) and sleep architecture (duration of light, deep, and REM sleep).
2. Anxiety and Depression The Hospital Anxiety and Depression Scale (HADS) will evaluate anxiety (HADS-A) and depression (HADS-D) at Pre-1, POD 1, POD 2, and POD 3. Each subscale comprises seven items scored 0–3; scores ≥ 8 indicate probable anxiety or depression (23).
3. Postoperative Pain and Recovery Quality Pain intensity will be assessed using the Numeric Rating Scale (NRS, 0–10) at rest and during movement on POD 1 (24). Analgesic consumption and patient-controlled analgesia demands will be recorded. Recovery quality will be evaluated on POD 1 using the 15-item Quality of Recovery (QoR-15) questionnaire (scores 0–150, higher scores indicating better recovery) (25).
4. Quantitative EEG Parameters (1) Absolute power: The integral of the power spectral density (PSD) over respective frequency bands (delta 1–4 Hz, theta 4–8 Hz, alpha 8–12 Hz, beta 12–25 Hz, and gamma 25–45 Hz). (2) Peak frequency: The frequency at which maximum PSD occurs within each band.

### 2.8 Safety evaluation

Safety monitoring will be conducted throughout the hospitalization period. Adverse events (AEs) and serious adverse events (SAEs)–defined per ICH-GCP guidelines (events resulting in death, life-threatening conditions, significant disability, prolonged hospitalization, or congenital anomalies)–will be meticulously documented and monitored until resolution.

Potential taVNS-related AEs include:

(1) Dermatological reactions (contact dermatitis, local paresthesia, electrode site irritation);

(2) Cardiovascular effects (hypotension, hypertension, bradycardia, tachycardia);

(3) Neurological symptoms (dizziness, headache);

(4) Respiratory events (oxygen desaturation); and

(5) Gastrointestinal disturbances (nausea, vomiting).

Serious adverse events will be reported to the ethics committee, principal investigator, and relevant regulatory authorities within 24 h per institutional policy. An independent Data Monitoring Committee (IDMC) will conduct scheduled safety reviews at 50% enrolment and biannually thereafter, focusing on SAE rates without efficacy analysis to preserve the overall type I error rate.

### 2.9 Data collection and management

Outcome and safety variables will be systematically collected across three distinct phases: preoperative, intraoperative, and postoperative. During the preoperative phase, demographic data, medical history, surgical information, laboratory values, and baseline assessments, including AIS, HADS, NRS for pain, and QoR-15, will be recorded at the enrolment visit immediately preceding the initial taVNS session. In the intraoperative phase, data on the duration of anesthesia and surgery, tracheal intubation time, estimated blood loss, fluid infusion volume, medication administration, and continuous EEG parameters will be meticulously documented. Postoperatively, the AIS and HADS will be assessed in the evenings of POD 1, 2, and 3. Additionally, the QoR-15 and NRS pain scores, both at rest and during movement, will be evaluated on the morning of POD 1, alongside the documentation of clinical outcomes and any adverse events.

All data will be systematically recorded using standardized case report forms (CRFs) and subsequently double-entered into an encrypted electronic database by two independent research assistants. A random sample comprising 10% of the CRFs, stratified according to the enrolment sequence, will undergo verification against the original medical records. The data manager will be responsible for generating queries related to missing or inconsistent values, with the principal investigator overseeing their resolution. In compliance with institutional policy and ICH-GCP guidelines, original CRFs will be securely stored in a locked cabinet with restricted access for a minimum duration of 5 years. The electronic data will be hosted on hospital servers, safeguarded by SSL encryption, automated backups, and role-based access control, ensuring access is limited to authorized study personnel.

### 2.10 Sample size calculation

The primary outcome is the incidence of POSD on POD 1, defined as an AIS score ≥ 6. Based on our internal pilot study (*N* = 20; taVNS *n* = 10, sham *n* = 10), the incidence of POSD (AIS ≥ 6) was 20% in the taVNS group compared to 45% in the sham group. Sample size was calculated using PASS 15.0 (NCSS, LLC) for a two-sided chi-square test comparing two independent proportions, with the following parameters: P1 (sham) = 0.45, P2 (taVNS) = 0.20, α = 0.05 (two-sided), power = 0.80. This calculation indicated a need for 52 evaluable participants per group. Accounting for an anticipated 10% dropout rate based on comparable perioperative neuromodulation trials, the final target enrollment is 116 participants (58 per group).

### 2.11 Statistical analysis

Statistical analyses will be performed using SPSS Statistics 26.0 (IBM Corp.) and R 4.4.0 (R Foundation). MATLAB R2018b (MathWorks, Natick, MA) will be employed for the preprocessing and spectral analysis of EEG data. Normality will be assessed using the Shapiro-Wilk test and Q-Q plots. Continuous variables will be presented as mean ± standard deviation or median (interquartile range), and categorical variables will be shown as frequencies and percentages.

The primary outcome (incidence of AIS ≥ 6 on POD 1) will be analyzed using the chi-square test to compare proportions between the taVNS and sham groups. The effect size will be expressed as an absolute risk reduction with a 95% confidence interval. Linear mixed-effects models will analyze repeated measures, including (1) AIS total scores (Pre 1 to POD 3); (2) HADS-A and HADS-D (as two separate scales, Pre 1 to POD 3); (3) objective sleep parameters from actigraphy; and (4) EEG spectral power across five bands (delta, theta, alpha, beta, and gamma) at six time points (T0–T5). Models include fixed effects for group, time, group × time interaction, and random intercepts for subjects. For repeated clinical outcomes, Bonferroni correction will be applied across four key endpoints: HADS-A, HADS-D, AIS trajectory, and objective sleep parameters from actigraphy, yielding a significance threshold of α = 0.0125 (0.05/4). For EEG spectral analyses, given the exploratory nature and high dimensionality (5 frequency bands × 6 timepoints), the Benjamini–Hochberg procedure will control the false discovery rate at q < 0.05.

A three-step approach will be used to identify predictors of POSD: (1) Univariate screening (*P* < 0.10) for 30 EEG variables (5 bands × 6 timepoints); (2) LASSO regularization (10-fold CV) to select independent predictors; (3) Multivariable logistic regression reporting ORs and 95% CIs.

The main analysis will adhere to the intention-to-treat methodology. To address missing data, multiple imputation via chained equations will be utilized, generating 20 imputations under the assumption that data are missing at random. Sensitivity analyses will incorporate pattern-mixture models to account for mechanisms where data are missing not at random. The per-protocol analysis will exclude cases with significant protocol deviations. Effect sizes will be reported as Cohen’s d, alongside 95% confidence intervals.

## 3 Discussion

This study investigates the clinical efficacy and underlying mechanisms of taVNS in alleviating POSD after gynecologic laparoscopic surgery by modulating brain rhythm stabilization, employing a randomized controlled trial design. POSD frequently occurs after gynecologic surgery, heightening the risk of postoperative pain, cognitive dysfunction, and cardiovascular events, thereby extending recovery time. Current treatments like benzodiazepines and melatonin focus on symptomatic relief without addressing the neurophysiological regulation of the sleep-wake cycle, and they offer limited effectiveness in managing complex, multi-dimensional issues synergistically. Cortical rhythmic activity serves as the core neurophysiological mechanism regulating the sleep-wake cycle; surgical stress and anesthetic agents can disrupt the power balance of cortical slow waves and fast waves, leading to fragmented sleep architecture. This study explores the innovative use of taVNS, a non-invasive neuromodulation technique, for postoperative sleep management by leveraging the central role of cortical oscillations in sleep homeostasis, aiming to offer a novel multi-target therapeutic strategy.

The association between EEG characteristics and POSD establishes a physiologically targetable endpoint for perioperative neuromonitoring. In a prospective cohort study, Dai et al. (26) demonstrated that reduced sleep spindle density in frontotemporal channels during surgery independently predicted POSD in elderly patients undergoing orthopedic procedures, confirming that specific EEG oscillatory patterns serve as predictive biomarkers for postoperative sleep disruption. Prior work establishes that intraoperative EEG signatures are modifiable targets rather than mere predictors of postoperative sleep quality. In gynecologic laparoscopy specifically, enhancing frontocortical beta and gamma power through esketamine administration reduces POD 1 sleep disturbance incidence (1). Similarly, elevated intraoperative gamma-band activity correlates with sleep protection in breast cancer surgery, suggesting a cross-surgical class effect (27). These convergent findings indicate that perioperative cortical oscillatory states are therapeutically accessible, providing the mechanistic premise for non-pharmacologic neuromodulation.

Accumulating evidence indicates that taVNS exerts therapeutic effects through modulation of cortical oscillatory activity (28, 29). An animal study found that taVNS can significantly improve the power spectrum of the delta band, reduce the power spectrum of the β band in EEG signals of insomnia model rats (30), and inhibit the excited state of the cerebral cortex in insomnia model rats. Furthermore, EEG reveals that taVNS normalizes alpha-band activity in the prefrontal cortex of patients with chronic pain, indicating reduced cortical hyperarousal (31). Randomized sham-controlled trials indicate that taVNS significantly improves sleep quality scores (Cohen’s *d* = 1.2), enhances slow-wave sleep continuity, and boosts daytime functioning in patients with chronic insomnia (32). Studies have found that taVNS can increase frontal delta power and right-lateralized delta asymmetry during resting states, suggesting enhanced homeostatic sleep pressure (18). However, its use for acute POSD after gynecologic surgery remains unexplored.

In this study, subjective sleep quality was evaluated utilizing the Athens Insomnia Scale (AIS), a rigorously validated instrument characterized by strong psychometric properties, which has been widely employed in numerous investigations examining postoperative sleep quality (33–35). Nevertheless, subjective scales are susceptible to recall bias and provide no information on sleep architecture; therefore, objective sleep parameters will be acquired concurrently. Given the practical constraints of conducting standard polysomnography (PSG) during the perioperative period, we will employ the HUAWEI WATCH GT2 smartwatch for objective sleep monitoring (validated against PSG: sensitivity 95.9%, accuracy 87.3%) (36) to obtain continuous physiological parameters, including sleep efficiency and wake after sleep onset. Moreover, taVNS’s influence on autonomic tone and possible decrease in perioperative anxiety (measured by the HADS-A subscale) might indirectly enhance sleep quality, thus breaking the cycle of sleep disturbances, increased pain, and prolonged recovery. Multidimensional scales such as the QoR-15 and NRS effectively indicate clinical symptom improvement and intervention efficacy in postoperative patients.

In addition, preoperative intervention can enhance the tolerance of the central nervous system to anesthetic interference (37), while the early post-anesthesia emergence period represents a critical window for rhythm reshaping (12); therefore, a dual-timepoint strategy combining preoperative and immediate postoperative interventions may be more effective. The non-invasive and easy-to-administer nature of taVNS renders this dual-timepoint approach clinically feasible.

This study makes three significant contributions. Theoretically, it integrates taVNS with perioperative neuroplasticity theory to enhance postoperative sleep management. By focusing on cortical rhythmic activity as the central mechanism, this approach transcends pharmacological methods that merely offer symptomatic relief without restoring neurophysiological homeostasis, thereby transitioning from passive sedation to active state reconstruction. Methodologically, the study employs a randomized, double-blind, sham-controlled design. The sham protocol involves titrating the current to the threshold of tingling sensation, followed by immediate cessation, while keeping the device powered to ensure blinding. For assessment, a comprehensive three-dimensional evaluation framework is constructed, incorporating subjective sleep outcomes, objective sleep parameters, and mechanistic measures. This framework facilitates correlation analysis between cortical oscillatory dynamics and sleep outcomes.

From a methodological perspective, the intervention protocol employed in this study is designed to adhere to standard taVNS parameters. The stimulation will be administered to the left cymba conchae, a region abundant in auricular vagal branches, at a frequency of 20 Hz with a pulse width of 250 μs. These parameters were selected based on previous efficacy studies related to chronic insomnia and cortical oscillation research, aiming to optimize the balance between tolerability and effective activation of the vagus nerve-thalamus-cortex pathway (32, 38). The sample size calculation is informed by internal pilot data, indicated a reduction in the incidence of POSD, defined as AIS score of 6 or higher, from 45% in the sham group to 20% in the taVNS group–an absolute risk reduction of 25 percentage points. To achieve 80% statistical power for detecting this difference in proportions at a two-sided significance level of α = 0.05, while accounting for a 10% dropout rate, a sample size of 58 participants per group will be required.

This study is subject to several limitations. To begin with, the single center design might restrict how widely the findings can be applied, since local patient traits like age distribution, initial sleep condition, and the complexity of surgeries could influence the intervention’s effectiveness. Nevertheless, the single-center setting enables standardization of surgeons, anesthesia protocols, and postoperative ward environments, thereby reducing confounding from institutional practice variations that frequently obscure treatment effects in multicenter perioperative trials. Secondly, our sample size estimation relies on internal pilot data. With a total of 116 participants, the study lacks sufficient power for conducting subgroup analyses, detecting rare adverse events, or making stratified comparisons based on age, operative duration, or anesthetic dosage. Thirdly, the primary observation window of 3 days may not capture longer-term effects on chronic sleep disturbances, delayed cognitive function, or sustained autonomic regulation.

Even with these limitations, the potential significance of this study is remarkable. Should taVNS be effective, it may offer a safe and economical non-drug approach to managing sleep disturbances after gynecologic surgery, fitting well within the multi-faceted symptom management of ERAS protocols. Even if null, the mechanistic EEG data and careful sham-controlled design would inform parameter selection, biomarker reliability, and the feasibility of perioperative neuromodulation trials. This research could uncover how cortical oscillatory activity regulates POSD, offer new insights into the role of the vagus nerve-thalamus-cortex pathway in perioperative care, and enhance comprehension of interactions between neural, autonomic, and sleep systems.
